# THE IMPACT OF GRANDCHILD PROBLEMS ON CORESIDENTIAL GRANDMOTHERS’ SITUATIONAL APPRAISALS

**DOI:** 10.1093/geroni/igad104.1141

**Published:** 2023-12-21

**Authors:** Alexandra Jeanblanc, Carol Musil

**Affiliations:** Case Western Reserve University, Cleveland, Ohio, United States; Case Western Reserve University, Cleveland, Ohio, United States

## Abstract

The lives of coresidential grandmothers and their families are often complicated by grandchild health, behavioral, and educational problems Using data from a nationwide sample of 342 American grandmothers living with and raising grandchildren, we examine the effect of grandchild physical, behavioral, and educational problems on co-residential grandmothers’ appraisals of: how positive their living situation is for themselves and for their grandchildren and their self-appraised stress and reward related to living with and caring for grandchildren. Participants lived with 1-7 grandchildren under the age of 18 (mean=1.82, SD 1.04), and grandchild problems were dichotomized on the basis of whether they were reported for any co-residential grandchild. We ran independent t-tests comparing grandchild problems against participants’ responses to the 4 situational appraisals (measured on a 10 point visual analog scale). The presence of grandchild health problems did not affect grandmother’s appraisal of their own living situation or their caregiving reward, but was significantly associated with a lower mean appraisal of how positive their living situation was for their grandchildren (8.39vs8.80) as well as higher reported caregiving stress. Grandchild behavioral problems were significantly associated with lower appraisals of grandmother’s own living situation (6.69vs7.53), grandchild living situation (8.45vs8.91), lower caregiving reward (7.36vs8.31)and higher caregiving stress (8.09vs6.81). Educational problems were significantly associated with lower appraisals of grandmother’s own living situation (6.70vs7.34), grandchild living situation (8.18vs8.93), lower caregiving reward (7.41vs8.06)and higher caregiving stress (8.18vs7.06). These analyses show the complex interaction between grandchild problems and grandmother perceptions of their own and their grandchildren’s’ lives.

